# Responsiveness and the minimal clinically important difference of the Chelsea Critical Care Physical Assessment tool (CPAx) in critically ill, mechanically ventilated adults: a study protocol for a prospective, multicentre, cohort study

**DOI:** 10.1136/bmjopen-2025-102374

**Published:** 2025-09-18

**Authors:** Sabrina Eggmann, Michelle Paton, Christa Villinger, Scott Bradley, Tahnee Hellings, Ashlea Hills, Philipp Venetz, Tessa Broadley, Anaïs Charles-Nelson, Carol Hodgson

**Affiliations:** 1 The Australian and New Zealand Intensive Care Research Centre, School of Public Health and Preventive Medicine, Monash University, Melbourne, Victoria, Australia; 2 Department of Physiotherapy, Alfred Health, Melbourne, Victoria, Australia; 3 Department of Physiotherapy, Inselspital, Bern University Hospital, Bern, Switzerland; 4 Department of Physiotherapy, Monash Health, Clayton, Victoria, Australia; 5 Department of Intensive Care Medicine, Inselspital, Bern University Hospital, University of Bern, Bern, Switzerland

**Keywords:** INTENSIVE & CRITICAL CARE, Adult intensive & critical care, REHABILITATION MEDICINE, Exercise

## Abstract

**Introduction:**

The use of invasive life support in patients with a prolonged critical illness clearly saves lives but carries substantial risks, including intensive care unit-acquired weakness (ICUAW) and long-term disability. Early mobilisation might improve outcomes, yet the evidence is conflicting and complicated by the lack of a responsive outcome measurement to detect change in critically ill patients’ physical function and activity. The Chelsea Critical Care Physical Assessment tool (CPAx) is a valid and reliable instrument for patients at risk of ICUAW. However, its ability to measure change over time (responsiveness) and the minimal clinically important difference (MCID) have not yet been rigorously investigated.

**Methods and analysis:**

The primary objective of this prospective, international, multicentre, longitudinal cohort study is to investigate responsiveness and to establish the MCID of the CPAx during the ‘intensive care unit (ICU) period’, from ICU baseline to ICU discharge, and ‘hospital period’, from ICU to hospital discharge. Adults with any critical illness who are mechanically ventilated for at least 72 hours, expected to remain in ICU (≥48 hours) and being treated by a physiotherapist are eligible for study inclusion. Functional measurements, including the CPAx and a global rating of change (GRC) scale, will be collected during routine physiotherapy. Responsiveness will be evaluated primarily using the GRC as an anchor to distinguish changed from unchanged/deteriorated patients (criterion validity). As such, the magnitude of change will be analysed with receiver operating characteristics. Additionally, construct validity will be explored with correlation coefficients and effect sizes to confirm/reject a priori formulated hypotheses. MCID will be investigated with anchor-based and distribution-based methods. We plan to recruit 120 patients across three sites in Australia and Switzerland.

**Ethics and dissemination:**

Ethical approval has been obtained from each local ethics committee (Canton of Bern, Switzerland (2024-00346), Monash Health, Australia (HREC/106143/MonH-2024-438474(v3)), the Alfred, Australia (490/24)). The results will be disseminated through international/national conferences, peer-reviewed journals and social media. The high quality, rigorous testing of the CPAx could benefit researchers, clinicians and patients.

**Trial registration number:**

NCT06419699.

**STRENGTHS AND LIMITATIONS OF THIS STUDY**

- Rigorous COnsensus-based Standards for the selection of health Measurement INstruments (COSMIN) methodology is applied to investigate the Chelsea Critical Care Physical Assessment tool in a large, international, multicentre cohort of critically ill adults with a prolonged intensive care unit (ICU) stay.
- Responsiveness and minimal clinically important difference are obtained for two clinically relevant, patient-centred periods, but major functional changes are conceivable in between.
- The burden for patients is kept minimal by using data from routine care.
- Change is estimated by clinicians and might not reflect patients’ perception of change.
- Attrition bias for the ‘hospital period’ might result from ICU death or transfers to other hospitals limiting generalisability.

## Background

 Survival after a prolonged critical illness has improved,^1 2^ but survivors frequently report poor physical, cognitive, mental and/or social health.^3^,^7^ This condition, termed post intensive care syndrome,^8^ might be driven by muscle loss and weakness^9^ whereby patients lose an average of 14% muscle mass within the first week of an intensive care unit (ICU) admission.^10^ ICU-acquired weakness (ICUAW) is associated with weaning failure, increased mortality and healthcare costs,^11^ as well as persisting physical impairments up to 5 years.^12 13^ Early mobilisation might improve survivors’ health.^14 15^ However, most studies are underpowered, single-centre and at risk of bias, lacking valid and responsive measurement instruments^16 17^ or detailed intervention-reporting such as the frequency, intensity, type or timing of activity achieved.^18^ Moreover, the largest trial to date found a signal of harm with high-intensity exercise, particularly in patients with diabetes.^19 20^ A responsive measurement tool for critically ill patients at risk of ICUAW is urgently needed to ensure reliable and robust results from early mobilisation trials.

The Chelsea Critical Care Physical Assessment tool (CPAx) has strong measurement properties for critically ill patients’ physical function and activity.^21^ It is a 10-item tool—rated on a 6-point Guttman scale—that includes the hallmark features of ICUAW, including respiratory function, cough, functional mobility and muscle strength.^22 23^ The CPAx score ranges from 0 (completely dependent) to 50 (independent in the measured constructs) without major floor and ceiling effects at ICU baseline to ICU and hospital discharge.^24^ The tool was predictive of the discharge destination, return to home and back to work in small, single-centre studies.^22 25 26^ Trained clinicians can score the CPAx in 5 min based on a standard physiotherapy session, or retrospectively from medical records if appropriate information is reported.^27 28^ Thereby, the burden for this vulnerable patient population is kept low. However, despite the increased use of the CPAx across the world,^29^,^32^ responsiveness has only been evaluated in small and selective samples.^2933^,^35^ Moreover, the minimal clinically important difference (MCID), relevant for sample size calculation, has not yet been established for critically ill patients at risk of ICUAW. This limits the usability of the CPAx in future clinical trials and the evaluation of patients’ progress in clinical practice. Rigorous investigation of responsiveness and the MCID of the CPAx is therefore urgently required. Additionally, it would be useful to investigate the predictive validity in a larger, international sample.

## Methods and analysis

### Objectives

This study, with an international population of critically ill adults with a prolonged ICU stay, aims to

1. Investigate the responsiveness of the CPAx, that is, its ability to detect change over time, from ICU baseline (study inclusion) to ICU discharge (‘ICU period’: primary outcome) and from ICU to hospital discharge (‘hospital period’: secondary outcome).
2. Establish the MCID of the CPAx for the ‘ICU and hospital period’.
3. Assess the predictive validity of the CPAx score at ICU baseline for the duration of mechanical ventilation and ICU length of stay, and at ICU discharge for the hospital discharge destination.

### Design

This prospective, international, multicentre, observational, longitudinal cohort study will be performed in accordance with the Declaration of Helsinki and Good Clinical Practice guidelines. The methodology is based on the COnsensus-based Standards for the selection of health Measurement INstruments (COSMIN) guideline^36^ and the protocol written according to the Strengthening the Reporting of Observational Studies in Epidemiology statement.^37^ An international, multicentre trial with a large sample of the target population across continents is required for the urgently needed outcome measurement to perform rigorous clinical trials in this field.

### Setting

This study will be conducted in two tertiary hospitals in Melbourne, Australia (Alfred Health, Monash Health) and one university hospital in Bern, Switzerland (Inselspital). Together, these hospitals include the full range of critical illness, including medical, surgical, trauma and burn units. The CPAx is routinely evaluated in clinical practice in Switzerland.^24 28^ Physiotherapists in Australian hospitals receive in-person CPAx training from the primary investigator^28^ prior to the start of the study to ensure reliability between centres.

### Patient and public involvement

The study protocol was initially written without patient and public involvement. Feedback on our protocol was provided by Hugh Burrill, who has a lived experience of a prolonged critical illness period (box 1).

Box 1 **Commentary from Hugh Burrill who has a lived experience of a prolonged critical illness**

- As a former patient, I fully support a study which seeks to validate clinically meaningful changes in physical status over time in patients admitted into ICU and their subsequent hospital stay. As this study does not involve any additional interventions but instead utilises data that is gathered during the provided physiotherapy, I feel that inclusion into this study should be readily accepted by patients and their family.
- One of the overriding feelings I experienced when being transferred from ICU and during my time in the hospital wards was the realisation of how significantly my mental and physical health had deteriorated since having the cardiac arrest. As a consequence, I was very motivated to seek treatments and activities that would improve those. I was always keen to get a sense of any quantifiable improvement in my physical (and mental) health. Therefore, if the CPAx tool were used to provide timely feedback to patients during their therapy as to their physical improvement, this would have been welcomed and motivating information. It would also have been comforting to know that the physiotherapy I was receiving was following approaches proven to be effective and guided by measurements using validated tools such as the CPAx.
- In thinking about my own experience and the level of disorientation and mental confusion I experienced while in ICU and in the hospital ward, I can imagine that it would be difficult for patients to rate their own perceived change in function and activity.

CPAx, Chelsea Critical Care Physical Assessment tool; ICU, intensive care unit.

### Participants

The target population for future clinical trials is critically ill patients with an increased risk for a prolonged ICU stay and ICUAW.^17^ Accordingly, adults (aged 18 years or older) with an acute critical illness of any origin who are receiving mechanical ventilation for at least 72 hours, who are seen by a physiotherapist and who are expected to remain in the ICU for a further 48 hours are eligible for study inclusion. This ensures a sufficient timeframe to detect a real change in physical function and activity. Exclusion criteria are imminent death (not expected to survive to hospital discharge), second or subsequent ICU admission for this hospital admission, transfer from an external ICU (with ICU stay of >72 hours) or a primary neurological diagnosis at ICU admission (ie, of the central nervous system including stroke, intracerebral haemorrhage, traumatic brain injury, spinal cord injury) as the CPAx is not validated in this cohort. We will further exclude patients who have a known pregnancy, those who lived in a care facility before their ICU admission (ie, with severe pre-existing mental or physical disability) or patients who need to be excluded by local ethical regulations.

### Procedures

There are no interventions in this observational study. Treating ICU physiotherapists at each site will screen potential participants during their daily clinical practice from Monday to Friday, maintaining a secure local log for all patients screened with reasons for study exclusion. Consent procedures follow local ethical requirements ranging from a waiver of consent to written informed consent.

Physiotherapy in the ICU is safe.^38^ Consequently, physiotherapy is routinely provided to all critically ill patients at the three participating sites. The chosen outcome measurements (table 1)^2239^,^43^ provide minimal burden for patients as they are based on routine physiotherapy interventions and can be scored even if mobilisation may be contraindicated. As such, physiotherapists complete the measurements as part of routine treatment, with documentation in the electronic health record. In case a patient is being discharged on a weekend (and not seen by a physiotherapist), the closest measurement to their discharge is recorded. Additionally, a global rating of change (GRC) scale for the ‘ICU and hospital period’ will be assessed by the treating physiotherapist.

**Table 1:** Outcome measurements and data collection schedule

Outcome measurement	Description	Source data	ICU baseline*	ICU discharge†	Hospital discharge‡
Chelsea Critical Care Physical Assessment tool (CPAx)^22^	Physical function and activity rated on 10 items from 0 to 5 with a maximal score of 50 indicating independence	Routine physiotherapy	x	x	x
Global rating of change (GRC) scale^39^	Physiotherapists rating on patients’ change for ‘physical function and activity’ using a seven-point scale: ‘(1) very much improved; (2) much improved; (3) little improved; (4) no change; (5) little deterioration; (6) much deterioration; (7) very much deterioration’	Treating physiotherapist		x ‘ICU period'	x ‘hospital period’
ICU Mobility Scale (IMS)^40^	Mobility level on a scale from 0 (nothing) to 10 (walking independently)	Routine physiotherapy	x	x	x
Medical Research Council Sum Score (MRC-SS)^41^	Muscle strength in major muscles of the upper and lower limbs with a maximal summed score of 60 (full strength), less than 48 points indicates ICUAW	Routine physiotherapy (observation if unable to follow commands)	x	x	x
Richmond Agitation-Sedation Scale (RASS)^42^	Level of sedation and/or cooperation with 0 meaning alert and calm, +4 combative and −5 unarousable	Medical records	x	x	x
Modified Iowa Level of Assistance Scale (mILOA)^43^	Required assistance in functional task for six items rated from 0 (independent) to 6 (not testable)	Routine physiotherapy	x	x	x
Demographic, diagnostic, discharge data	Includes age, gender, body mass index, Acute Physiology and Chronic Health Evaluation (APACHE) II, age-adjusted Charlson Comorbidity Index (CCI), admission diagnosis, length of mechanical ventilation, ICU and hospital length of stay, mortality and discharge destinations	Medical records	x	x	x

* Within 72–144 hours after ICU admission.

† Within 24 hours before or after ICU discharge.

‡ Before hospital discharge.

ICU, intensive care unit; ICUAW, intensive care unit-acquired weekness.

### Responsiveness

Responsiveness is defined as the ‘ability to detect change over time’ and is considered an aspect of validity by the COSMIN guideline.^44^ Accordingly, criterion validity of CPAx change scores will be investigated with the GRC as a gold standard to distinguish patients with change (GRC 1–3) from those unchanged or deteriorated (GRC 4–7). These definitions are based on the clinical rationale that patients are at their lowest level of physical function and activity during the need for invasive mechanical ventilation (ICU baseline). No change or deterioration at ICU discharge is therefore equivalent to continued invasive life support or death, justifying their combination as an undesirable outcome. Patients who die will therefore be allocated the worst possible CPAx score of 0 indicating their inability to survive without invasive life support. In addition, construct validity will be explored using prospective hypotheses for each period about the expected magnitude of change as well as the expected correlation for change-scores between the CPAx and the other outcome measurements. The hypotheses are based on a previously published conceptual model^24^ reflecting on the overlapping constructs of the CPAx with the other measurement instruments and considering potential floor and ceiling effects for each period. Table 2 lists prospective hypotheses as well as the statistical methods for analysis.

**Table 2:** A priori hypotheses for the expected magnitude of change scores (construct validity)

	Measured constructs	Hypotheses ‘ICU period’	Hypotheses ‘hospital period’
CPAx	Movement, function of muscles and respiratory system, mobility	Effect size >0.8 (calculated as mean change/SD average)	Effect size >0.8 (calculated as mean change/SD average)
IMS	Level of mobility	Positive, high correlation between CPAx and IMS (r>0.7)	Positive, moderate to high correlation between CPAx and IMS (r>0.6)
MRC-SS	Muscle function: used as reference standard for ICUAW (MRC-SS<48)	Positive, moderate to high correlation between CPAx and MRC-SS (r>0.6)	Positive, moderate correlation between CPAx and MRC-SS (r=0.4–0.7)
mILOA	Movement and mobility	Negative, moderate to high correlation between CPAx and mILOA (r>−0.6)	Negative, moderate to high correlation between CPAx and mILOA (r>−0.6)

CPAx, Chelsea Critical Care Physical Assessment tool; ICU, intensive care unit; ICUAW, intensive care unit-acquired weekness; IMS, ICU Mobility Scale; mILOA, Modified Iowa Level of Assistance Scale; MRC-SS, Medical Research Council Sum Score; r, Spearman’s rank correlation coefficient.

### Minimal clinically important difference

The MCID corresponds to the smallest, clinically meaningful change over time—a particularly relevant criterion to interpret efficacy of treatments.^44^ Despite a lack of consensus, the COSMIN guideline recommends using anchor-based approaches with a GRC over distribution-based methods that commonly rely on measurement error or heterogeneity of a population.^44^ A new systematic approach has been proposed to triangulate the MCID from anchor-based methods by using a range of distribution-based indicators.^45^ This approach has the advantage of considering clinical importance, the magnitude of the change and the variability of the measurement focusing on improvement as is appropriate for our population. Finally, we will determine the robust clinically important difference (RCID)^45^ to account for potentially major changes between the ‘ICU and hospital periods’. To this end, major change is defined as GRC 1–2 and minimal change as GRC 3.

### Predictive validity

Hypotheses testing will be used to confirm earlier research about the predictive validity of the CPAx, including the exploration of sensitivity and specificity for a return home after hospital discharge.^25^ The following hypotheses are tested:

- The CPAx score at ICU baseline will negatively correlate with the duration of mechanical ventilation and the ICU length of stay (low to moderate correlation of −0.3 to −0.6).
- The CPAx at ICU discharge increases in a ranked order for three hospital discharge destinations (undesirable (ie, death or external transfer)≤in-patient rehabilitation≤home).

### Data management

Project data will be handled with the utmost discretion and only accessible to authorised personnel who require the data to fulfil their duties within the scope of their clinical practice and/or this research project. To protect all data from unauthorised or accidental disclosure, from alteration, deletion, copying and theft, paper-based data collection forms will be secured in a locked facility approved by the participating institution and/or electronic-based data collection in password-protected local area network computer drives supported by institution’s information technology services. To this end, all local project leaders will be trained in data controlling and entry. Ultimately, solely non-identifiable original study data will be collected and managed with Research Electronic Data Capture (REDCap) tools hosted and managed by Helix (Monash University, Australia).^46^ REDCap is a secure, web-based application designed to support data capture for research studies, providing (1) an intuitive interface for validated data entry; (2) audit trails for tracking data manipulation and export procedures; (3) automated export procedures for seamless data downloads to common statistical packages and (4) procedures for importing data from external sources.^46^

### Statistical analysis

Descriptive statistics will summarise population variables, single measurements and change scores using mean±SD, median (IQR) or number (n) with percentages (%) as appropriate. We will further calculate the percentages of minimally (GRC 3), majorly (GRC 1–2) and unchanged patients (GRC 4–7), as well as floor (minimum score) and ceiling effects (maximum score), of which the last are considered acceptable if <15%.^47^

Responsiveness by criterion validity will be analysed with receiver operating characteristic (ROC) statistics with an expected area under the ROC (AUROC) curve of >0.6 for the ‘ICU period’ (primary outcome) and ‘hospital period’ (secondary outcomes). We plan to conduct sensitivity analyses to explore the robustness of our results by excluding patients who died from the unchanged group, and in case of relevant outliers, by excluding them. Patients with a stay of more than 90 days will be measured at day 90 as their ICU discharge and excluded for hospital discharge. We will perform subgroup analyses by country due to potential differences in healthcare systems and discharge policies. Responsiveness by construct validity (secondary outcome) will be analysed as described in table 2 and considered excellent if ≥75% of the a priori hypotheses can be accepted and as moderate if 50%–74% are accepted.^47^

MCID and RCID will be triangulated using anchor-based methods with Youden’s Index on distribution-based indicators as described by Malec and Ketchum.^45^ Accordingly, CPAx change scores and their correlation with the anchor will be computed, whereby a Spearman correlation coefficient of >0.35 is considered unacceptable.^45^ In case of an unacceptable (>0.35) or poor (<0.7) correlation, we plan to use the ICU Mobility Scale (IMS) as an anchor which has an anchor-based MCID of 3 points for sensitivity analysis.^48^ Distribution-based indicators of interest are SE of measurement (SEM)^24^; ½ baseline (pre-treatment) SD; 1.96×SEM; Reliable Change Index=2.77×SEM^24^ and 1 SD (baseline).^45^ SEM is a measure of reliability and will therefore be used from previous research with a similar cohort.^24^ We will perform subgroup analyses by country.

Predictive validity will be analysed with Spearman’s rank correlation and ROC curve coordinates on sensitivity and specificity to establish the optimal cut-off point for a return to home.^25^

The analyses will be conducted with IBM SPSS Statistics (V.29) with a set two-sided α of 0.05 without any adjustments for multiple testing. Participants with missing outcome data will be analysed based on their availability for each period (pairwise exclusion). There will be no imputation of missing data.

### Sample size calculation

The COSMIN guideline recommends a sample size between 30 and 50 patients in the smallest group to investigate responsiveness via criterion validity.^49^ Based on our previous data,^24^ we expect the smallest group to be the ‘unchanged’ group, whereby approximately 26% of patients will not change when assuming a change of 6 points to be clinically relevant,^33^ yielding a sample size of n=115.

Solely considering survivors with an expected lack of change in 16%, we calculated that a sample of 97 survivors is required to demonstrate an AUROC of 0.8 (null hypothesis 0.6) with a power of 0.8 and an α of 0.05 (MedCalc on 26 December 2022). This is in line with the recommendation of ≥100 participants for a construct validity approach.^49^ We therefore aimed to include 120 participants (40 per centre). Based on our previous research,^24^ we do not expect any major missing data as all data are routinely documented at each site.

## Ethics and dissemination

### Ethics

This study has received ethical approval from the local ethics committees of the Canton of Bern, Switzerland on 30 April 2024 (Project-ID 2024-00346), Monash Health, Australia on 29 August 2024 (HREC/106143/MonH-2024-438474(v3)) and the Alfred, Australia on 4 September 2024 (Project No: 490/24). The study was prospectively registered on clinicaltrials.gov on 13 May 2024 (NCT06419699). The first patient was recruited on 23 May 2024 in Switzerland. We expect recruitment and follow-up to be completed by the end of August 2025.

The CPAx has been specifically developed for patients who are critically ill, which is a particularly ethically vulnerable population. However, a measurement instrument must be evaluated in the target population.^36^ While the CPAx is a valid and highly reliable tool for this population, a healthy population would suffer from ceiling effects which would render the score redundant to assess change. We therefore took every measure to keep the burden for the target population low by choosing routine assessments and the collection of standard healthcare data only. Patients who are not being treated by physiotherapists will not be included. Any identifiable or re-identifiable data will solely be stored locally with disseminated data being non-identifiable to protect patients’ privacy. Confidentiality will be maintained in accordance with local legislations for privacy and the use of healthcare data. Data transfer agreements or research agreements have been signed as appropriate by participating institutions.

The CPAx is not a patient-reported outcome measure. Moreover, ICU survivors generally report difficulty in processing their functional recovery at ICU discharge and rely on their caregivers to set adequate treatment goals.^50^ The perspective of the clinical physiotherapist seems therefore an appropriate anchor for change and should reduce missing data as patients often cannot remember their ICU stay.^51^

### Dissemination

The results from this study will be disseminated at national and international conferences, in peer-reviewed journals with an appropriate target audience as well as through social media to reach a wide audience and other relevant stakeholders, including patients and their families. Finally, results of this project will inform future, international, randomised controlled trials investigating the effect of early mobilisation on critically ill patients’ physical function and activity, with the CPAx as the primary outcome using the MCID for sample size calculation.
