# Supplementary material for: Responsiveness and the minimal clinically important difference of the Chelsea Critical Care Physical Assessment tool (CPAx) in critically ill, mechanically ventilated adults: a study protocol for a prospective, multicentre, cohort study
Source: BMJ Open. 2025 Sep 18;15(9):e102374. doi: 10.1136/bmjopen-2025-102374 (PMC12458801; doi:10.1136/bmjopen-2025-102374)
Supplement: Supplementary file 1 [file bmjopen-2025-102374.reviewer_comments.pdf]

PEER REVIEW HISTORY

BMJ Open publishes all reviews undertaken for accepted manuscripts. Reviewers are asked to complete a checklist review form and are provided with free text boxes to elaborate on their assessment. These free text comments are reproduced below.

ARTICLE DETAILS

Title (Provisional)

Responsiveness and the minimal clinically important difference of the Chelsea Critical Care Physical Assessment tool (CPAx) in critically ill, mechanically ventilated adults – a study protocol for a prospective, multicentre, cohort study

Authors

Eggmann, Sabrina; Paton, Michelle; Villinger, Christa; Bradley, Scott; Hellings, Tahnee; Hills, Ashlea; Venetz, Philipp; Broadley, Tessa; Charles-Nelson, Anaïs; Hodgson, Carol

---

VERSION 1 - REVIEW

**Reviewer**	**1**
**Name**	**Van Aswegen, Helena**
**Affiliation**	**University of the Witwatersrand, Physiotherapy**
**Date**	**19-Jun-2025**
**COI**	**None**

I would like to congratulate the authors on a well-written protocol. The justification for the study is sound and I agree that it is important to establish the responsiveness and MCID for the CPAx tool as it is often used by physiotherapists as part of patient care in ICU throughout the world.

I have some comments that I would like the authors to consider:

- Abstract: no mention is made of the other tools namely the ICU Mobility Scale, MRC-SS, and mILOA (all presented in Table 1) - why these are used and how data obtained with these tools will be compared to CPAx.

- Objectives: The rationale for the objective related to predictive validity is not covered in the Background to the study. This objective is also not mentioned in the Abstract.

- Exclusion criteria: The criteria for patients admitted with multiple orthopaedic injury, acute spinal cord injury, abdominal trauma requiring extensive and repeated laparotomy procedures needs to be more explicit e.g. will these patients be included or excluded from

the study? Will patients with burn injury be included? What percentage of total body surface area burns will be considered not suitable for participation?

- Procedures: Is the CPAx used routinely in patient care by the physiotherapists who work in the participating hospitals? If not, what training intervention will be put in place to ensure that all physiotherapists are experienced in administering the CPAx when the study commences?

- MCID: Will MCID be reported for the study cohort as a whole or will cognisance of the different admission criteria be taken and MCID reported for each admission category? If it will be reported for each admission category, is a sample size of 120 participants adequate to achieve accurate reporting?

- Statistical analysis: It is not clear how the data obtained from the MRC-SS and the mILOA will be used in the analysis. Please provide this information.

- ORCID: Please provide the ORCID ID for Christa Villinger.

I look forward to reading the revised manuscript for this noteworthy study.

VERSION 1 - AUTHOR RESPONSE

Reviewer #1

I would like to congratulate the authors on a well-written protocol. The justification for the study is sound and I agree that it is important to establish the responsiveness and MCID for the CPAx tool as it is often used by physiotherapists as part of patient care in ICU throughout the world.

We thank the reviewer for taking the time to review our manuscript and are grateful for the constructive and valuable feedback to strengthen our manuscript. We answer all comments below.

I have some comments that I would like the authors to consider:

- Abstract: no mention is made of the other tools namely the ICU Mobility Scale, MRC-SS, and mILOA (all presented in Table 1) - why these are used and how data obtained with these tools will be compared to CPAx.

We thank the reviewer for raising this point that gives us the opportunity to explain our reasoning. The main objective of this study is to establish responsiveness. According to the COSMIN guideline, there are several approaches to achieve this, including criterion validity and / or construct validity of change scores. In our statistical analysis plan, we specified that the primary outcome will be criterion validity. This will be analysed with receiver operating characteristic (ROC) statistics for the ‘ICU period’ as described in the abstract. To ensure robustness of results as there is no current consensus on the best approach for responsiveness (i.e., both criterion validity and construct validity are recognised), we also want to analyse our results using a construct validity approach. Thus, we will investigate

change-scores as a secondary outcome. To this end, we have created a-priori hypotheses about the expected magnitude of change (effect sizes) as well as about the expected correlation between the CPAx and the other outcome measurements (i.e., IMS, MRC-SS, mILOA). All hypotheses are listed in Table 2. In total, we have 8 hypotheses, 4 for each period (ICU and hospital), we therefore cannot list them in the abstract. However, to clarify, we have made the following changes to the abstract:

"Responsiveness will be evaluated primarily using the GRC as an anchor to distinguish changed from unchanged/deteriorated patients (criterion validity). As such, the magnitude of change will be analysed with receiver operating characteristics. Additionally, construct validity will be explored with, correlation coefficients and effect sizes of to confirm/reject a-priori formulated hypotheses."

We also slightly revised the manuscript improve clarity (page 8, lines 197-203):

"In addition, construct validity will be explored using prospective hypotheses for each period about the expected magnitude of change as well as the expected correlation for change-scores between the CPAx and the other outcome measurements. The hypotheses are based on a previously published conceptual model[24] reflecting on the overlapping constructs of the CPAx with the other measurement instruments and considering potential floor and ceiling effects for each period. Table 2 lists prospective hypotheses as well as the statistical methods for analysis."

- Objectives: The rationale for the objective related to predictive validity is not covered in the Background to the study. This objective is also not mentioned in the Abstract.

We appreciate this comment as indeed a rationale was lacking for this secondary outcome. We have revised this by adding two sentences to the background section. In short, our rationale is to confirm results from previous small, single-centre studies in a larger, international cohort. The added sentences / revisions are (page 5, lines 116-117 & 125-126):

"The tool was predictive of the discharge destination, return to home, and back to work in small, single-centre studies."

"Rigorous investigation of responsiveness and the MCID of the CPAx are therefore urgently required. Additionally, it would be useful to investigate the predictive validity in a larger, international sample."

We also clarified in the abstract that the listed objective (responsiveness) is the "primary outcome". This should indicate to readers that secondary outcomes are likely without distracting them with less relevant, secondary objectives (page 3, line 54):

"The primary objective of this prospective, international, multi-centre, longitudinal cohort study is to investigate responsiveness and to establish the MCID of the CPAx…"

- Exclusion criteria: The criteria for patients admitted with multiple orthopaedic injury, acute spinal cord injury, abdominal trauma requiring extensive and repeated laparotomy procedures needs to be more explicit e.g. will these patients be included or excluded from the study? Will patients with burn injury be included? What percentage of total body surface area burns will be considered not suitable for participation?

The reviewer asks some excellent questions. The CPAx has been developed for all patients admitted to an ICU with a critical illness regardless of the cause. It is therefore not limited to a certain patient group with the exception of a neurological admission diagnosis, as it has not yet been validated in that cohort. Our aim was therefore to include a very broad sample of patients with a critical illness to allow use of the CPAx in as many patients as possible. The three recruiting centres mirror that choice as they cover the full range of critical illness including medical, surgical, trauma, and burn units (page 6, line 150). To answer your specific question, yes, a patient with multiple orthopaedic injury, abdominal trauma (or thorax trauma) or a burn injury would be included if no exclusion criteria were present. In some of these cases, the relevant exclusion criteria might be "not expected to survive". This will not depend on a cut-off value for example on total body surface area (TBSA) for burn patients, which is in line with a previous study from the CPAx developer (Corner et al. 2015) that included burn patients regardless of their TBSA.

We have clarified the section about participants (page 7, lines 162-165):

"Accordingly, adults (aged 18 years or older) with an acute critical illness of any origin who are receiving mechanical ventilation for at least 72 hours, who are seen by a physiotherapist, and who are expected to remain in the ICU for a further 48 hours are eligible for study inclusion."

In contrast, an acute spinal cord injury would be excluded because we consider this a neurological admission diagnosis. We have added this diagnosis to the list of exclusions (page 7, lines 169-170):

"…or a primary neurological diagnosis at ICU admission (i.e., of the central nervous system including stroke, intracerebral haemorrhage, traumatic brain injury, spinal cord injury) as the CPAx is not validated in this cohort."

- Procedures: Is the CPAx used routinely in patient care by the physiotherapists who work in the participating hospitals? If not, what training intervention will be put in place to ensure that all physiotherapists are experienced in administering the CPAx when the study commences?

The reviewer raises an excellent point. In consequence, we have added a statement about the training for physiotherapists, see page 6, lines 150-153):

“The CPAx is routinely evaluated in clinical practice in Switzerland.[24 28] Physiotherapist in Australian hospitals receive in-person CPAx training by the primary investigator based on [28] prior to the start of the study to ensure reliability between centres.”

- MCID: Will MCID be reported for the study cohort as a whole or will cognisance of the different admission criteria be taken and MCID reported for each admission category? If it will be reported for each admission category, is a sample size of 120 participants adequate to achieve accurate reporting?

The idea to report MCID for each admission category is excellent. However, we agree, our sample size is too small for this. Accordingly, we did not plan and do not intend to perform a separate analysis. Our aim is to validate responsiveness of the CPAx for a broad ICU cohort to allow functional scoring for all of them. We acknowledge that there might be heterogeneity; however, the CPAx has been repeatedly confirmed as valid in various ICU populations. The only planned subgroup analysis for the MCID is by country. We have added this to the manuscript (page 10, line 265):

“We will perform sub-group analyses by country.”

- Statistical analysis: It is not clear how the data obtained from the MRC-SS and the mILOA will be used in the analysis. Please provide this information.

We have formulated multiple hypotheses for analysis to confirm construct validity of change scores, all are listed in Table 2. In particular, we will use Spearman’s rank correlation coefficient (r) to assess the correlation of change-scores between the CPAx and the IMS, MRC-SS, and mILOA. The expected magnitudes of the correlation are also listed in Table 2 (r = Spearman’s rank correlation coefficient). Hypotheses are based on the measured constructs for each assessment (IMS, MRC-SS, mILOA) and how they overlap with the constructs of the CPAx. They further consider potential floor or ceiling effects for the measurement period as these might lead to smaller changes.

	**Measured constructs**	**Hypotheses ‘ICU period’**	**Hypotheses ‘hospital period’**
**CPAx**	Movement, function of muscles and respiratory system, mobility	Effect size > 0.8 (calculated as mean change / SD average)	Effect size > 0.8 (calculated as mean change / SD average)
**IMS**	Level of mobility	Positive, high correlation between CPAx and IMS (r > 0.7)	Positive, moderate to high correlation between CPAx and IMS (r > 0.6)
**MRC-SS**	Muscle function: used as reference standard for ICUAW (MRC-SS <48)	Positive, moderate to high correlation between CPAx and MRC-SS (r > 0.6)	Positive, moderate correlation between CPAx and MRC-SS (r = 0.4 to 0.7)
**mILOA**	Movement and mobility	Negative, moderate to high correlation between	Negative, moderate to high correlation between

		CPAx and mILOA (r > -0.6)	CPAx and mILOA (r > -0.6)

We have clarified the manuscript with the following sentence (page 8, lines 197-203):

"In addition, construct validity will be explored using prospective hypotheses for each period about the expected magnitude of change as well as the expected correlation for change-scores between the CPAx and the other outcome measurements. The hypotheses are based on a previously published conceptual model[24] reflecting on the overlapping constructs of the CPAx with the other measurement instruments and considering potential floor and ceiling effects for each period. Table 2 lists prospective hypotheses as well as the statistical methods for analysis."

Page 10, lines 254-256:

"Responsiveness by construct validity (secondary outcome) will be analysed as described in Table 2 and considered excellent if ≥75% of the a-priori hypotheses can be accepted and as moderate if 50-74% are accepted."

- ORCID: Please provide the ORCID ID for Christa Villinger.

We have added her ORCID ID, which is https://orcid.org/0009-0008-3856-7105

I look forward to reading the revised manuscript for this noteworthy study.

Thank you very much for your insightful review, we appreciate your perspective and believe this has improved our manuscript.

VERSION 2 - REVIEW

**Reviewer**	**1**
**Name**	**Van Aswegen, Helena**
**Affiliation**	**University of the Witwatersrand, Physiotherapy**
**Date**	**02-Sep-2025**
**COI**	

I am satisfied that the authors answered each of my queries in an appropriate manner. I wish them all the best with their multicenter trial.
